# “Football is a boys’ game”: children’s perceptions about barriers for physical activity during recess time

**DOI:** 10.1080/17482631.2017.1379338

**Published:** 2017-10-17

**Authors:** Maria Martínez-Andrés, Raquel Bartolomé-Gutiérrez, Beatriz Rodríguez-Martín, Maria Jesus Pardo-Guijarro, Vicente Martínez-Vizcaíno

**Affiliations:** ^a^ Centro de Estudios Sociosanitarios, Universidad de Castilla-La Mancha, Cuenca, Spain; ^b^ Facultad de Enfermería, Universidad de Castilla-La Mancha, Albacete, Spain; ^c^ Facultad de Terapia Ocupacional, Logopedia y Enfermería, Talavera de la Reina, Universidad de Castilla-La Mancha, Toledo, Spain; ^d^ Facultad de Educación, Universidad de Castilla-La Mancha, Cuenca, Spain; ^e^ Facultad de Ciencias de la Salud, Universidad Autónoma de Chile, Talca, Chile

**Keywords:** Focus groups, drawing, physical activity, children, school, qualitative research

## Abstract

The aim of the study was to know the factors that influence boys and girls’ perceptions for performing physical activity during playground recess from their own perspective. Ninety-eight schoolchildren aged 8–11 years from five schools from Cuenca (Spain) participated in 22 focus groups and carried out 98 drawings following the socioecological model as a theoretical framework. A content analysis of the transcripts from the focus groups and drawings was carried out by three researchers. Results showed that, in spite of boys and girls identified same barriers, there were gender differences in their perceptions. Gender socialization was the key as central category and helped to understand these differences. Boys preferred play football and this sport had a monopoly on the recess space. Weather was a barrier for boys. Girls and boys, who did not play football, were relegated to peripheral areas and lack of materials was a barrier for them. Teachers were a barrier for all children who did not play football. Thus, in order to promote recess physical activity, researchers, teachers and educational policy makers should take into account gender socialization and promote inclusive non-curricular physical activity in schools.

Physical activity during childhood has been associated with physical, psychological and social benefits (Eime, Young, Harvey, Charity, & Payne, 2010; Janssen & Leblanc, 2010). There is substantive evidence suggesting that children engaged in at least 60 minutes of moderate-to-vigorous intensity physical activity on most days of the week have health benefits that might be sustained through adolescence and into adulthood (Currie et al., 2012; Janssen & Leblanc, 2010; Reilly & Kelly, 2011). However, the prevalence of children who do not meet these recommendations is growing at an alarming rate in some countries (Ng et al., 2014).

Schools are the ideal environment to promote active behaviours. During school hours, recess offers children some opportunities to get the minimum levels of recommended physical activity (Beighle, Morgan, Le Masurier, & Pangrazi, 2006; Huberty et al., 2011; Ridgers, Stratton, & Fairclough, 2006) as well as chances to interact with their peers (Blatchford, Baines, & Pellegrini, 2003).

Current research on physical activity during recess has predominantly focused on objectively quantifying the amount of physical activity or testing the effectiveness of school-based interventions to increase children’s physical activity during recess (Parrish, Okely, Stanley, & Ridgers, 2013; Ridgers, Salmon, Parrish, Stanley, & Okely, 2012). In addition, other studies using qualitative approaches have primarily focused on conceptualizations (related to potential factors that influences physical activity) from an adult perspective (i.e., parents and teachers) rather than a child’s (Darbyshire, 2005).

Three previous studies have explored children’s barriers for physical activity during recess from their own perspective using qualitative approaches (Parrish, Yeatman, Iverson, & Russell, 2012; Pawlowski, Tjørnhøj-Thomsen, Schipperijn, & Troelsen, 2014; Stanley, Boshoff, & Dollman, 2012); however, only one was focused on the gender perspective (Pawlowski et al., 2014), and none have been carried out in Mediterranean countries. These studies identified barriers related to the individual (skills), social environment (lack of facilities to play or teacher support, bullying), physical environment (lack of spaces/equipment, adverse weather conditions) and organizational environment (school policy, recess duration) without considering gender differentiation. Currently little is known about the underlying conceptualizations behind children’s preferences for recess physical activity (barriers and enablers) due to gender differences (Ridgers et al., 2012). Although we did not have any pre-establish hypothesis regarding gender differences, it is a significant variable in environmental and development studies (Gill, 2002) and should be taken into account when research focuses in physical activity preferences and motivations (Chalabaev, Sarrazin, Fontayne, Boiché, & Clément-Guillotin, 2013).

Engaging physical activity is a complex matter (Finegood, 2011) because exercise-related behaviours are influenced by multiple factors at different levels, as Bronfenbrenner’s socioecological model depicts (Bronfenbrenner, 1999). Therefore, the present study is built following Bronfenbrenner’s socioecological model.. The aim of the study was to find out the factors that influence boys and girls’ perceptions for performing physical activity during playground recess. In order to meet this goal we propose the following research questions: (1) Is the recess time a moment or space to physical activity at the school? (2) What are the enablements or barriers to practicing physical activity during the recess time? and (3) Which factors or processes have an influence schoolchildren’s perceptions about enablements or barriers?

## Methods

### Participants

This qualitative study is part of the MOVI-2 project (Martinez-Vizcaino et al., 2012), a recreational and non-competitive (i.e., handball, hockey, steal the fling sock, dance) after-school physical activity intervention that involved children from 20 schools of the province of Cuenca (Spain). The participants were 8-to 11-year-old schoolchildren in the 4th and 5th grades of Primary Education (www.movidavida.org). Parents and teachers also participated by completing some questionnaires regarding sleep habits, children’s satisfaction with the program or academic achievements. The study protocol was approved by the Clinical Research Ethics Committee of the Virgen de la Luz Hospital.

To the present qualitative study, a subsample of 98 schoolchildren (36 boys and 62 girls) from MOVI-2 project was invited to participate. The inclusion criteria were: schoolchildren, both sexes, belonging to different socioeconomic status and from schools located in urban and rural settings (participating schools from MOVI-2) (Table 1).

**Table 1.** Characteristics of participants.

	Boys	Girls	Total	
Urban place	28	36	64	98
Rural place	8	26	34	
Socioeconomic Status	Low		2,5%	
	Middle		85%	
	Upper		12,5%	

Meetings were held with parents to inform them of the aims and methods of the study, and to request their informed consent. We strongly encouraged them to consider their children’s opinion before signing the consent. All children agreed to participate, except two girls whose parents did not agree to be recorded. The data collection was completed when the participants stopped providing new information and the saturation point was reached (Giacomini & Cook, 2000). Boys’ speeches reached saturation point before girls’, thus research continued but focusing on boys only from now on.

### Instruments and procedure

In order to meet the objective, a qualitative approach was designed combining two complementary techniques: (1) analysis of the children’s drawings about their environment (Morrow, 2001) and (2) focus groups (Taylor & Bogdan, 1984). Each session was conducted by two researchers, one of them acted as moderator and the other as observer. Between February and April 2011, 22 sessions were conducted, 11 of which involved five children and the remaining ones involved four children either because we lacked their parent’s consent or they missed the appointment. Lasting on average 40 minutes each. All sessions took place in the children’s school in order to make them in a familiar environment for children, and were recorded on audio and video.

All sessions began by requesting the children to make a personal drawing, consisting of a map with the places they usually go on during weekdays (MCDougall, Schiller, & Darbyshire, 2004). Children were given 20 minutes to complete the drawing (Darbyshire, 2005). After completing the drawings, children were gathered in focus groups supported by the use of a script, following different levels of the sociological model, (Table 2) and the personal drawings. Focus groups lasted 20 minutes.

**Table 2.** Focus groups script.

Main question	Probing questions
What kind of activities do you practice in the playground?	Do you practice the same activities every day?
	Do you like them?
	Are these activities organized?
What kind of games do you play?	Traditional games, sports, electronic devices…
Where do you practice these activities and games?	What are these places like?
	Green space, recreational space, leisure and sport facilities, weather…
With whom do you practice these activities and games?	Boys, girls, course, classroom, teachers…

Transcripts from the focus groups recordings were sorted and organized accordingly, and three qualitative methodology experts analysed this data following a three-step approach taking into account age and gender. First, the data were divided into codes, then these codes were grouped into categories and subcategories and, finally, the categories were organized into a central topic. The process was a continuous comparative method in which coding and clustering discrepancies were discussed with the research team until all of the members agreed (Giacomini & Cook, 2000).

The F4 Software tool was used to transcribe the focus groups and Atlast.TI 5.0 for processing the data (drawings and text analysis).

Drawings and focus groups were used to triangulate data. To triangulate data is to use two or more methods or techniques, in order to validate the information provided by the different participants, increasing the credibility and trustworthiness of the results. The use of drawings and maps helped the researchers to find out more about the main places where children usually went, also showed the facilities available for them at their school, and helped them verbalise their opinions during the focus groups (Giacomini & Cook, 2000). This way, researchers analysed the correspondences between the oral discourses and the drawings. If there were discrepancies between them for any participants, the speaker’s discursive contributions were not taken into account.

### Rigour

Several strategies were used to guarantee a rigorous analytical approach. The credibility and the trustworthiness of the findings was enhanced through the selection of the participants: urban and rural settings, both sexes, different school year and different ages. The drawings were used to encourage children to express their opinions and experiences, thus producing as a result a relaxed atmosphere that invited children to talk. In order to find about the adaptability and suitability of the techniques to the characteristics of the participants, four trial sessions were carried out in a meeting room at the University of Castilla-La Mancha- Cuenca. In these sessions, the researchers checked different kinds of drawings (group or individual dynamic), different kinds of groups (only boys, only girls and mixed) and also refined the script with common words suitable for children. This session had the following results: individual drawings and mixed groups were the top options, but children had to be part of the same grade, leading to deeper and richer speeches. Finally, the coding was analysed with three levels of depth, by three separate researchers (Giacomini & Cook, 2000). After each level of coding had been completed, the researchers held a discussion to identify similarities and discrepancies about levels of coding or labels to identify the codes or categories. Although the discrepancies were minimum, they were solved by involving other members of the team.

## Results

**Figure 1.**
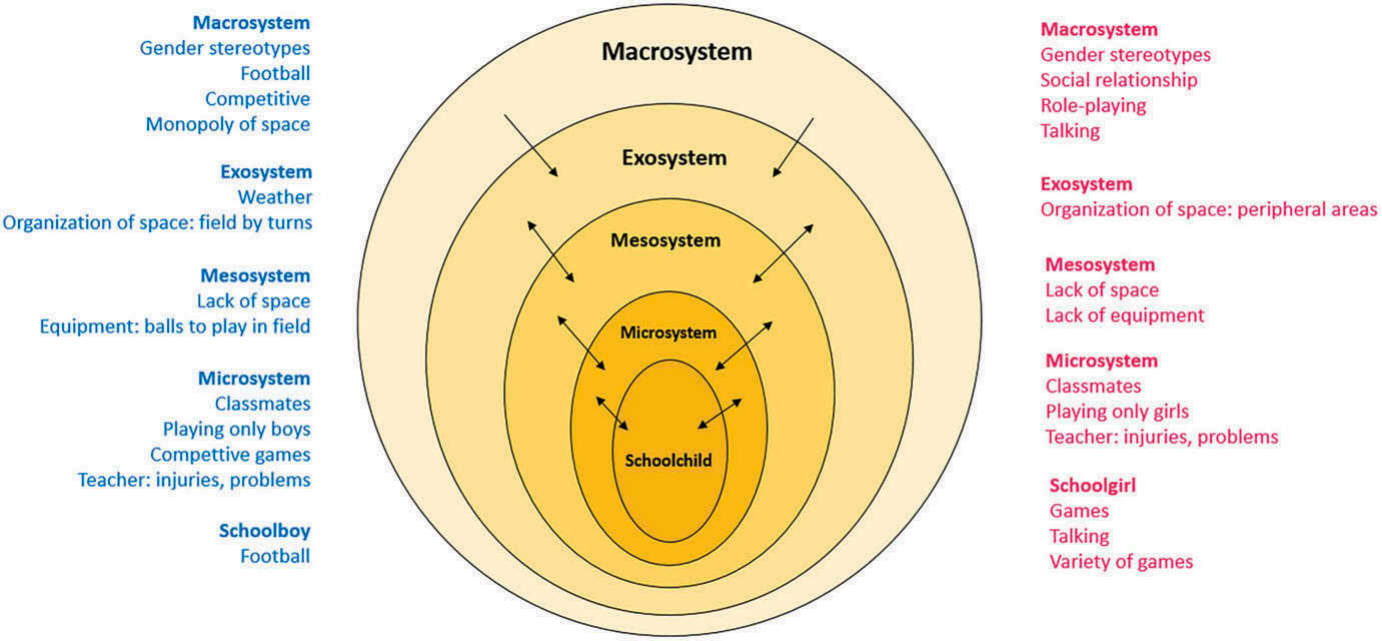
Levels socioecologial model. jpg.

After analysing the data, the factors that influence boys and girls’ physical activity during playground recess were based on five levels of influence following the socioecological model: the schoolchild and his or her biological characteristics (age, gender, skills, preferences); her or his peers and teachers (microsystem); conflicts and lack of space (mesosystem); playground organization, rules and weather (exosystem); and, finally, gender socialization (macrosystem) (Figure 1). Both boys and girls included aspects of these five levels of influence as barriers to physical activity, but we found some gender-based differences in their perceptions and importance, which we explain below.

### Schoolchild and biological characteristics

Individual level is influenced by age, gender, skills and preferences. While girls preferred games and some sports (basketball) during the recess time, boys only opted for sports, especially very competitive ones, and in particular football. Moreover, we found that girls frequently played non-active games, and they preferred to spend the recess time talking or doing role-playing games rather than performing physical activity:

Girls don’t play; we’re simply walking around the playground, talking…. (Girl)

We prefer football. (Boy)

Other individual aspects such as personal beliefs (their own consideration of being overweight, clumsy or not good at sports) influenced in children’s perceptions. Thus, some children, especially boys, believed that they were not good enough to play with other boys:

I’m bored because nobody passes me [the ball]. (Boy)

Most playground games developed by children were group-based and spontaneous, involving certain structure and rules, following their preferences and tastes. In order to overcome boredom, girls ended up participating in a greater variety of games than boys, who preferred to just play football during recess. Moreover, in the specific case of boys we found that the activities did not change throughout the year and that they usually preferred competitive sports and games:

I like to play football all the time. (Boy)

I get bored when I play the same game over and over. (Girl)

### Microsystem: peers and teachers

We found that the number of classmates had a direct impact in children’s choice of activities during the recess. Children attending a school would normally stay in the same class and see the same people throughout the years, sharing the same playtime partners too. So choices about the activities performed during the recess time required an agreement to be reached, especially in urban areas. However, in rural areas there were fewer children per class, so they shared playground games that involved a greater variety of participants such as children of different ages and grades:

When we get the whole class together…we can play hide-and-seek for a while. (Girl)

We play football with children in 4th grade, class A and B, and sometimes those in 3rd-grade. (Boy)

Interrelationships with classmates was another important factor. Being trusted by your peer group and being accepted by them had an important influence to the possibility of being chosen to participate in certain games and sports, in particular those competitive ones:

I don’t like playing football, it’s boring and because I’m bad at it, they [classmates] choose me last to play in their team and I’m always the goalkeeper. (Boy)

On the other hand, we found that girls and boys did not share activities. There were games only for boys and games only for girls. Both boys and girls also believed they would not get along with the opposite sex. Particularly, girls considered that boys were more aggressive, very competitive, cheats, ball-hogs and rude:

Girls play in a group, all together and we boys play against each other, but not against girls. (Boy)

We play separately, because boys and girls get on badly. (Girl)

Relationship with teachers is another factor affecting physical activity in children. These relationships were opportunistic and children only requested their involvement when they needed help because of injuries, had problems with other children or lacked the right equipment. Teachers would not tell children what activities to play; however, if there was a specific game that might raise any sort of issues amongst the children, the tendency would be to stop allowing it:

Previously teachers let us play football, but because some guys were throwing the ball so hard and hit others…and they fall down, sometimes we hit each other…and fall to the ground. (Boy)

### Mesosystem: lack of space and equipment and conflicts

**Figure 2.**
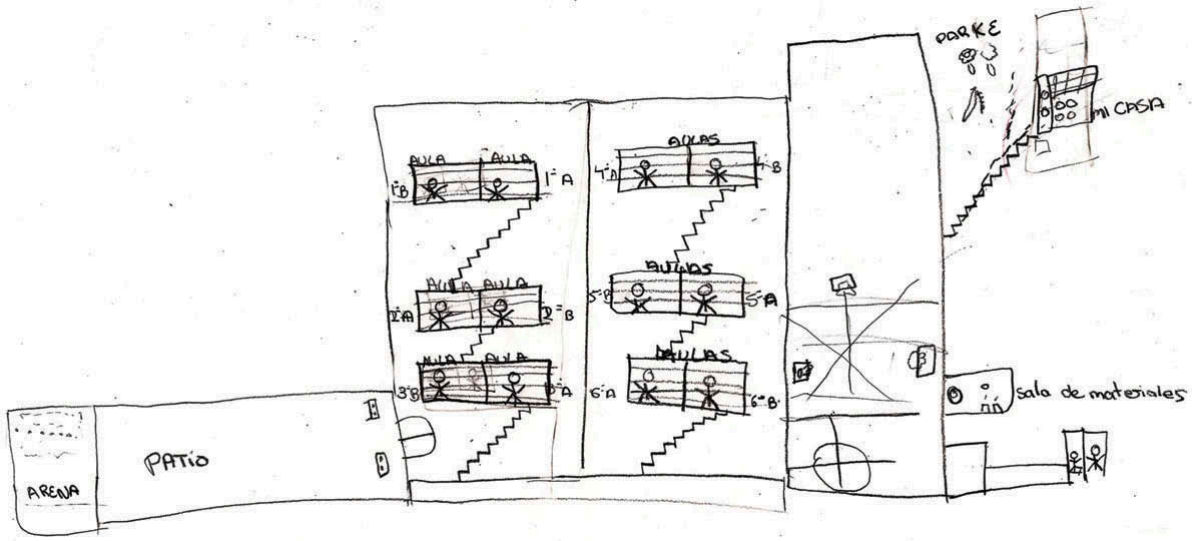
Girl, urban area.jpg.

**Figure 3.**
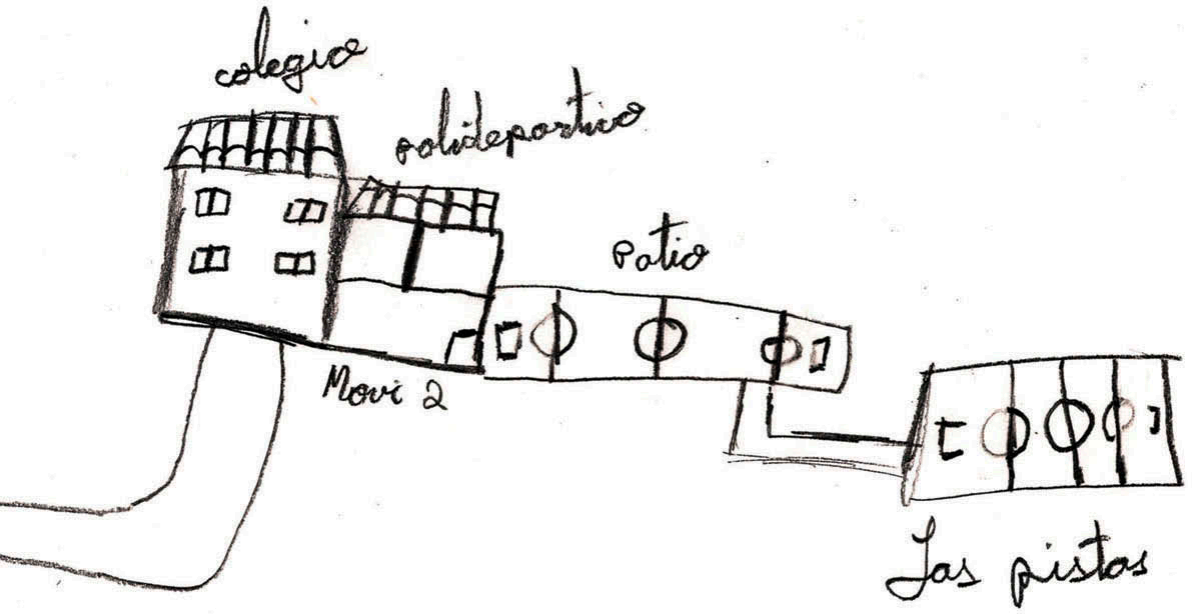
Boy, rural area.jpg.

Lack of space in the playground and lack of equipment and sports’ facilities were other factors that schoolchildren perceived as barriers to physical activity during the recess. In Spain, school playgrounds will usually have two differentiated areas (multisport field and free space), as shown in the drawings (e.g., Figures 2 and 3).

The playground was represented in their drawings as a relevant area, and as important because the school playgrounds will usually have two differentiated areas. Lack of space affected the type of games and their intensity: active or non-active. Nevertheless, some children tried to play their favourites games and looked for a way to perform them, even if they had to do it in a rudimentary way:

We play football where the pine trees are, the goal is between two pine trees. (Boy)

In general, schools would not provide any equipment to play games and children would bring their own from home, so if they forget the equipment they can not play those games and teachers would only lend equipment to play properly organized sports, such as football, in the designated areas:

They used to let us borrow things, for example skipping ropes, balls…but now they only do it for those whose turn it is to play on the sport field. (Girl)

### Exosystem: organization of space, rules and weather

In the absence of such space and equipment, teachers tried to establish rules for the use of space. For example, they allocated a specific day for the use of sport field to each school year. These turns allowed a better distribution of the space during recess:

We can’t fit 100 people in the same playground. (Boy)

The organization and operation during a working day at school influenced children’s physical activity. During recess time, activities were rarely organized by teachers and, if so, these were organized by days and grades. In both rural and urban schools, teachers divided the use of sports fields by school year and day of the week (e.g., on Monday children in 4th grade use the sport fields, on Tuesday children in 5th grade.) and these could affect the activities that children did during the recess time. Teachers gave children the chance to pick a sport (e.g., basketball, handball or football). The majority of boys would often prefer football, confining girls, less skilful boys and those not in their turn to use the sports field, to peripheral areas:

During the recess we play separately, boys play football and girls play other games. (Girl)

Thursday is the day when we can use the sports field and we can choose the game.: What kind of games do you choose? (Interviewer)

Football, basketball…but we always choose football. (Boy)

In addition to these aspects, we found that the weather, and especially bad weather conditions were barriers to practicing physical activity during recess time. Thus, when it would rain or snow, children did not go to the playground. In those cases, they spent the recess time in their own classroom and the type of games were sedentary due to the lack of space for active games. Boys seemed less happy with this because they were using the field most of the time:

When it rains, we play cards in the classroom, like today. (Boy)

### Macrosystem: gender socialization

The analysis of the speeches showed gender differences in the type of activities for boys and girls. The process of gender socialization had an influence in their choice and confirmed the existence of gender stereotypes. Thus, we observed that boys preferred competitive games and sports, while girls were more interested in games involving social interaction (talking, role-playing) and therefore they spent more time socializing. However, some girls also wanted to be part of the game boys were playing and they were allowed after their skills were tested accordingly:

It was the first time I played [football] and nobody passed me, they [boys] kicked me and threw me to the floor…after that I was playing every day and then they passed me the ball more. (Girl)

Doing games such as Pantene® hair [shampoo commercial] or mimic posh girls. (Girl)

These differences in the preferences resulted in a monopoly of the space by boys and the discrimination against girls and less-skilful boys. There could be two main reasons for this: boys chose competitive games such as football and the way teachers organized the space following boys’ preferences.

Because boys don’t give us to play football. (Girl)

Football is a boys’ game. (Girl)

## Discussion

This study bridges a knowledge gap on perceptions about barriers to practice physical activity during recess, taking into account gender differences and how these perceptions could help to understand why girls are less active than boys. As far as we know, this is the only Spanish study where gender differences from a children’s point of view were been taken into account. We believe this is also the first one conducted in a Mediterranean country and one of the firsts across Europe (Pawlowski et al., 2014).

The socioecological model has been adapted to identify those factors and processes that influence in children’s physical activity during recess. Taking into consideration the complexity of the school system and the diversity of relationships that happen in this setting, we have used the model to approach in depth a specific environment, the recess, where children spend time playing free-games among peers (Finegood, 2011; Hendrickx, Mainhard, Boor-Klip, Cillessen, & Brekelmans, 2016). Boys and girls identified barriers in all levels (schoolchild and his or her biological characteristics, microsystem, mesosystem, exosystem and macrosystem); however, those barriers did not affect them equally due to gender socialization, which is the key and central category of the analysis. This analysis has brought to light which gender socialization is the process that allows understanding the different perception of the barriers between them. Thus, this study shows how socialization in a specific gender system (macrosystem) has influence in the development of individual preferences of game, in the reproduction of gender stereotypes among peers, in the monopoly of the space, which boys do, as in the acceptance of this monopoly from teachers.

According to Bronfenbrenner (Bronfenbrenner, 1999), individual characteristics play an important role in understanding how similar environments are perceived in different ways by different people. In this sense, our study was identified differences in preferences and participation in games dependent on children’s gender and abilities. In this way, both girls and boys participated separately in playground games (Pearce & Bailey, 2011). Along the same lines, in other studies, girls spent more time in sedentary activities than boys during the recess time (Hallal et al., 2012; Martinez-Vizcaino et al., 2014; Ridgers et al., 2012). At the next level, individual characteristics influence how children are perceived and treated by their peers, that which produces a specific microsystem of relation among them. In our results, this microsystem of relationships during recess promotes a gender construction where sports and competitive games are associated with masculinity, while stillness relates to femininity (Blatchford et al., 2003; Chalabaev et al., 2013; Paechter & Clark, 2007; Slater & Tiggemann, 2010).

Ridgers (Ridgers et al., 2012) suggested that girls prefer activities to socializing. However, our study shows that gender stereotypes could be influenced the children’s preferences. In this regard, Pawlowski et al. (2014) reported that most girls avoided taking part in aggressive and competitive activities, although some of them would like to participate in games with boys, but the boys did not accept them as playmates. These results showed that girls who liked to participate in games and sports, had to demonstrate their skills to be accepted by boys. Most of the time, there is not an opportunity to show their skills in these kind of games and sports, mainly football. Thus, football became the most popular game during recess time for boys as in other studies (Loucaides, Jago, & Charalambous, 2009; Pawlowski et al., 2014; Ridgers, Stratton, Fairclough, & Twisk, 2007). Nevertheless, along the same line of other studies, our results show football was more than just a game, symbolizing differences in play activities, intensities and opportunities for active play between boys and girls (Pearce & Bailey, 2011; Skelton, 2000). In this sense, the barrier for girls and less skilful boys is not being aggressive players (which may also require specific interventions) but a rough playing style or tumble games.

Previous research has reported that the lack of space was perceived as a significant barrier for playing (Pawlowski et al., 2014; Ridgers, Fairclough, & Stratton, 2010; Ridgers et al., 2012), but our results showed that the conflicts caused by the use of space is perceived as a larger barrier, than the lack of it (Sallis et al., 2001). In this way, negotiation about the use of the space shapes like a mesosystem (Neal & Neal, 2013) where individual interests of schoolchildren, relationships among peers and relationships with teachers converge. Football was the most played activity for boys and required a larger space, which means that football players would take over the main areas of the schoolyard (Blatchford et al., 2003; Boyle, Marshall, & Robeson, 2003). In both our study and other research, teachers mediated in the conflicts caused by the lack of space (Pawlowski et al., 2014; Sallis et al., 2001; Willenberg et al., 2010) and organized it around football providing equipment only for boys who played it (Sallis et al., 2001; Willenberg et al., 2010). However, this solution caused the children who did not play football—usually girls and less skilful boys—to be relegated to peripheral areas and become forced viewers of others (Blatchford et al., 2003; Boyle et al., 2003; Ridgers et al., 2012; Thomson, 2005). In addition, being active or not playing football should have a negative impact in these schoolchildren -girls and less skilful boys- because of having the necessity of being accepted by their peers and being isolated if they do not play with them (Salvy et al., 2008). Thus, in spite of teachers attempting to avoid this type of conflicts and ostracism, they played such a neutral role that boys ended up imposing their preferences and did not permit that the rest of their classmates could benefit from their turn to use the sports field. This indirect influence of teachers has been previously showed in the classroom and called “the invisible hand” (Hendrickx et al., 2016). Our findings show that this influence is also present at the recess.

In order to facilitate that both boys and girls could play active and non-active games, school staff should reduce the importance of football during recess, providing alternative options and avoiding the monopolization of the space that this activity encourages, as suggested by other studies (Hyndman, Telford, Finch, & Benson, 2012; Loucaides et al., 2009; Ridgers et al., 2007). The management team and the teachers might play a pivotal role in the rational distribution of the physical space during recess (Hyndman et al., 2012; Pawlowski et al., 2014).

Our results showed that the conflicts regarding the rules about the use and distribution of the schoolyard, and the shortage of equipment (exosystem) were perceived as a barrier to practice physical activity, which was similar to previous studies (Parrish et al., 2013; Ridgers et al., 2012). Supporting playground games during recess would mean introducing non-curricular physical activity, inclusive games, and less or no equipment. Children should also be offered the possibility of performing several games at the same time, for different ages, abilities and preferences (Loucaides et al., 2009). Furthermore, these kinds of games allowed girls and boys to play together. Playground games, as opposed to sports, require less space and equipment, and the space can be distributed more easily amongst a greater number of children, offering a wider variety of games (Loucaides et al., 2009). However, our results indicate the solution to promote physical activity to avoid obesity is a complex issue (Finegood, 2011) where not only decent facilities and equipment or space organisation through task structure might be sufficient. Gender should also be taken into account in order to know whether this has an impact on motivation and participation or not (Chalabaev et al., 2013). Aydt and Corsaro (2003) found that boys participated in less stereotyped games or games considered to be more feminine when they could safeguard their masculine identity. Therefore, any intervention to promote cross-sex or less competitive games should take into account this need to ensure that gender identity is prevailed. Perhaps not taking these issues into consideration had an impact in the fact that children who participated in MOVI-2 intervention did not include this kind of activities in their daily games during the recess time.

A strength of this study is the use of a qualitative methodology in order to find out environmental perceptions as a barrier or facilitator of physical activity for children, from their own perspective. In addition, the use of triangulation facilitated the enrichment of the analysis and enhanced the credibility of the results (Giacomini & Cook, 2000). The development of both the script and the analysis were supported by the socioecological model adapted to a specific setting, which allowed multiple factors (enablers or barriers) to be considered across all the different levels within the model. Finally, the fact that 98 children (36 boys and 62 girls), from both urban and rural areas, were involved in the study, helped with its transferability and reproducibility.

However, there are several limitations. First, the age of the participants, 8–11 years old, adds complexity to the design and analysis of the focus groups. Second, there was a higher number of girls participating in the study because their discourse was more heterogeneity, and consequently their saturation point was reached later. Third, the current dominant culture promoting physical activity in schools and the mass media could be influencing children’s discourse by making them aware of the importance of being active through advertisements or cartoons (Leavy, Bull, Rosenberg, & Bauman, 2011). Triangulation was used to mitigate these limitations (Giacomini & Cook, 2000). The use of two techniques allowed us to gather more complete and reliable information as well as to provide different levels of depth when answering the research questions (Denzin, 1989; Giacomini & Cook, 2000). Previous studies using drawings have described this technique as a useful strategy to focus children’s thoughts on the issues that would be addressed later on in the focus group (Willenberg et al., 2010). Furthermore, the perspectives considered in this study are only from the children’s point of view and may differ from the teachers’ and parents’ perspectives. Nevertheless, our objective was to find out a children’s viewpoint, with the belief that they have their own opinions arising from their independent experiences outside of the adults’ perspectives (James & Prout, 2005).

## Conclusion

This study shows the importance of gender socialization in barriers to practicing physical activity and the prevalent rules in recess time. Gender socialization explains the monopoly of the space for boys, first by choosing football as the main game during the recess, and second, by imposing their preferences over girls and less skilful boys, in the distribution of space. In addition to this, the results highlighted that gender perspective is an important element that the researchers should keep in mind in the design and an accurate interpretation of the results and the qualitative methodology is a good choice to deeply understand the influences on children’s physical activity. Future research ought to take into account gender stereotypes to design studies to promote physical activity and obtain successful results that endure over time.
